# Fractionated Stereotactic Gamma Knife Radiosurgery for Large Brain Metastases: A Retrospective, Single Center Study

**DOI:** 10.1371/journal.pone.0163304

**Published:** 2016-09-23

**Authors:** Joo Whan Kim, Hye Ran Park, Jae Meen Lee, Jin Wook Kim, Hyun-Tai Chung, Dong Gyu Kim, Hee-Won Jung, Sun Ha Paek

**Affiliations:** 1 Department of Neurosurgery, Seoul National University College of Medicine, Seoul, Korea; 2 Department of Neurosurgery, Seoul National University Hospital, Seoul, Korea; National Cancer Institute, UNITED STATES

## Abstract

**Purpose:**

Stereotactic radiosurgery (SRS) is widely used for brain metastases but has been relatively contraindicated for large lesions (>3 cm). In the present study, we analyzed the efficacy and toxicity of hypofractionated Gamma Knife radiosurgery to treat metastatic brain tumors for which surgical resection were not considered as the primary treatment option.

**Methods and Materials:**

Thirty-six patients, forty cases were treated with Gamma Knife-based fractionated SRS for three to four consecutive days with the same Leksell frame on their heads. The mean gross tumor volume was 18.3 cm³, and the median dose was 8 Gy at 50% isodose line with 3 fractions for three consecutive days (range, 5 to 11 Gy and 2 to 4 fractions for 2 to 4 consecutive days). Survival rates and prognostic factors were analyzed.

**Results:**

The overall survival rate at one and two years was 66.7 and 33.1%, respectively. The median survival time was 16.2 months, and the local control rate was 90%. RTOG toxicity grade 1 was observed in 3 (8.3%) patients, grade 2 in 1 (2.7%) patient and grade 3 in 1 (2.7%) patient respectively. Radiation necrosis was developed in 1 (2.7%) patient. KPS scores and control of primary disease resulted in significant differences in survival.

**Conclusions:**

Our findings suggest that consecutive hypofractionated Gamma Knife SRS could be applied to large metastatic brain tumors with effective tumor control and low toxicity rates.

## Introduction

Metastatic brain tumors are the most common intracranial tumors, outnumbering primary brain tumors and occurring in up to 25–30% of cancer patients.[1] The natural course of these patients are approximately 1 month survival without any intervention.[2] Aside from chemotherapy, the treatment options for brain metastases include steroid medication, surgical resection, whole-brain radiation therapy (WBRT) and stereotactic radiosurgery (SRS). Corticosteroid treatment alone results in a median survival of 2 months; the median survival with WBRT is 4 to 6 months, while for surgical resection of a single lesion, the median survival is 9 to 14 months.[3–6] Brain metastases are often inoperable because of their location, multiplicity, comorbidity, and performance status.[7] SRS has resulted in local control rates of 71 to 96% and a median survival of 7 to 13.5 months in previous studies.[8–11] Traditionally, the indication for SRS has been a lesion diameter of 3 cm or less, and large size is a relative contraindication due to treatment-related toxicity.[12] The concept of fractionation was first introduced in radiotherapy to reduce the toxicity of treatment for large tumors. Similar efficacy and lower toxicity risk of fractionated radiotherapy compared with single dose SRS has been demonstrated in several studies.[13–15] Previous reports have only studied LINAC-based SRS; however, Gamma Knife-based SRS has been rarely reported.[16, 17]

In this study we analyzed the efficacy and toxicity of hypofractionated Gamma Knife radiosurgery in the treatment of large metastatic brain tumors. Our hypothesis was that hypofractionated radiosurgery has equal effectiveness and tolerable toxicity rates.

## Patients and Methods

Between January 2010 and July 2015, 70 patients with a total of 79 lesions of brain metastases were treated with Gamma Knife-based fractionated radiosurgery. The patient selection criteria for fractionation treatment instead of single fraction were lesions greater than 14 cm^3^ or lesions greater than 10 cm^3^ located in posterior fossa. Because it is known that lesions greater than 3 cm in diameter is relative contraindication for single fraction radiosurgery due to risk for edema after treatment.[12] Therefore, 25 patients with lesions greater than 14 cm^3^ and 11 patients with posterior fossa lesions with volume greater than 10 cm^3^ were included in the study. There were no patient exclusion criteria for number of metastases or previous radiation treatment. The patient and treatment characteristics are summarized in Table 1. Patients were treated with hypofractionated radiosurgery instead of surgery due to the location of the lesion, poor general condition (age, comorbidities) and the wishes of the patients. The median age of the patients was 56 years at the time of treatment; there were 16 men and 20 women. The histology of the primary tumors included lung cancer, breast cancer, colorectal cancer and others. A solitary metastasis was present in 17 patients, while multiple metastases occurred in 19. Patients with multiple metastases were treated with single fraction radiosurgery for other smaller lesions. Primary disease was controlled in 25 patients. The median Karnofsky performance status (KPS) was 90, ranging from 60 to 100. Recursive partitioning analysis (RPA) and graded prognostic assessment (GPA) were analyzed. The mean gross tumor volume was 18.3 cm^3^. All data were obtained from hospital chart and imaging study databases; the study was approved by the Institutional Review Board. (IRB no. 1508-084-695) Patients and relatives were informed about the role, limitation and toxicities of radiosurgery. The requirement for obtaining informed consent from the patients was waived because the study was only based on the information obtained as a part of routine clinical care and their medical records. All patients received IV dexamethasone 10 mg on admission, followed by a maintenance dose of 4 mg every 6 hours for 3 consecutive days until the end of radiosurgery; oral prednisolone was then tapered over one week but was prolonged if symptoms caused by peritumoral edema persisted. Prophylactic antiepileptic medication was not used.

**Table 1:** Patient characteristics and treatment parameters.

Characteristics	Value
Number of patients	36
Age (years)	
Median	56
< 65	27 (75)
≥ 65	9 (25)
Gender	
Male	16 (44.4)
Female	20 (55.6)
Primary cancer	
Non-small cell lung cancer	10 (27.8)
Breast cancer	10 (27.8)
Colorectal cancer	5 (13.9)
Renal cell carcinoma	2 (5.5)
Others^a^	9 (25)
Previous treatment	
None	22 (61.1)
WBRT	8 (22.2)
Radiosurgery	6 (16.7)
Number of metastasis	
1	17 (47.2)
2–3	12 (33.3)
>4	7 (19.5)
Primary disease	
Uncontrolled	21 (58.3)
Controlled	15 (41.7)
Karnofsky performance status	
Median	90
Range	60–100
RTOG RPA class	
1	6 (16.7)
Others	30 (8)
GPA score	
0–1	7 (19.4)
1.5–2.5	18 (50)
3–4	11 (30.6)
SRS dose (Gy)	
8 * 3 fractions	24 (66.6)
Others	12 (33.3)
Gross tumor volume (cm^3^)	
10–14	11 (30.6)
>14	25 (69.4)
Mean	18.3
Range	10.0–50.3
Prescribed tumor volume (cm^3^)	
Mean	21.2
Range	11.5–67.8
Covered ratio (%)	95–99

^a^ Other histology include: 2 Squamous cell carcinoma, 2 Ovarian cancer, 1 Melanoma, 1 Hematologic malignancy, 1 Ewing sarcoma, 1 Cervix cancer, 1 Unknown.

Leksell Gamma Knife-based radiosurgery was performed (Elekta Instrument AB, Stockholm, Sweden, Model Perfexion). The treatment plan utilized the Leksell Gamma Plan (version 8.3.1, 10.1.0, 10.1.1, Elekta Instrument) system with thin-section magnetic resonance imaging (MRI). T1–weighted images with gadolinium enhancement were used to determine the target volume. The radiosurgery isodose and marginal dose prescribed were initially determined based on the tumor volume calculated during dose planning, using the best-fit isodose method; the prescription volume covered 95 to 99% of the gross tumor volume (GTV). The treatments were designed to cover 50% of the maximal dose to the margins of the target in a single fraction. The mean prescribed tumor volume was 21.2 cm^3^. The main prescription dose was 8 Gy of 3 fractions for three consecutive days (range, 5 to 8 Gy and 2 to 4 fractions for 2 to 4 consecutive days), using the same Leksell frame on the heads of the patients. The radiation dose was calculated to biological equivalent dose (BED) using a/b ratio of 10 and single fraction equivalent dose (SFED) using Dq of 1.8 for analysis of dose related response. The fractionation radiation dose calculated to BED and SFED are summarized in Table 2.[18, 19]

**Table 2:** Fractionation radiation dose in BED and SFED.

Fractionation schedule	Cases (%)	BED_10_	SFED (Gy)
8 Gy x 3	24 (66.6)	43.2	20.4
10 Gy x 3	6 (16.6)	60	26.4
9 Gy x 3	2 (5.5)	51.3	23.4
5 Gy x 4	2 (5.5)	30	14.6
7 Gy x 3	1 (2.7)	35.7	17.4
11 Gy x 2	1 (2.7)	24.2	20.2

BED = Biological equivalent dose, SFED = Single fraction equivalent dose.

Follow up was typically conducted at one and three months after radiosurgery, followed by MRI examinations at 3-month intervals. Additional neuroimaging was obtained if neurologic signs or symptoms developed. The tumor volume was measured on the follow-up MRI scans as previously described. Local control failure was defined as an increase in tumor volume to >125% of that measured at the time of radiosurgery.[20] Distant control failure was defined as progression in the brain but not within the radiosurgical target volume. Radiation-related brain necrosis was thoroughly assessed using the T1/T2 mismatch on the MRI scan.[21] Although there is no clear criteria for defining radiation necrosis, we performed positron emission tomography (PET) imaging for those with high possibility.[22] Surgical resection was encouraged when clinical signs of cerebral herniation or imminent herniation developed in the context of a radiographic diagnosis of local tumor progression or radiation necrosis during the follow-up period. Treatment toxicity was also assumed when the Karnofsky performance status score decreased or when the neurologic signs or symptoms worsened, combined with a stable or decreasing contrast-enhancing lesion within the radiosurgical target volume with increasing peritumoral edema. Overall survival was defined as the interval between radiosurgery and the death of the patient. Kaplan-Meier survival plots were used to estimate the overall survival distributions. The log-rank test (level of significance, *p<*0.05) was used to assess differences in the overall survival distributions between the groups. The Cox proportional hazards model (level of significance, *p* 0.05) was used to adjust for covariates, which were categorically tested. All statistical analyses were performed using IBM SPSS Statistics, version 22.0.0.1.

## Results

The follow up period ranged from 1 to 51.9 months, with a median follow up period of 13.4 months. The one-year and two-year overall survival rates were 66.7 and 33.1%, respectively, with a median survival time of 16.2 months. The local tumor control rate was 90%. Twenty three had died, and thirteen patients survived to the last follow up. Sixteen patients died of systemic progression, and seven patients died of neurological deterioration. New metastatic brain lesions were found in eleven patients: four patients with a single lesion and seven patients with multiple lesions. These new lesions were treated with repeat GK SRS in four patients, WBRT in three patients, surgical removal in one and conservative management in three patients. Of these, four survived and seven died. The clinical course of the neurologic deficits is shown in Table 3. Symptoms such as headache, cranial nerve palsy and weakness were observed in 15 patients before treatment. Among these patients, 10 patients showed improvement, 4 patients remained stable and only 1 patient was aggravated. All follow-up was done within 1 or 3 months after treatment. There were no cases of newly developed neurological deficit after SRS.

**Table 3:** Clinical course of neurologic deficits after SRS.

	No. of patients	Follow up		
		Improved	Stable	Worse
**New deficit after SRS**	0			
**No symptoms**	21			
**Pre-existing symptoms**	15			
** Headache**	6	3	3	0
** Motor/sensory deficit**	7	5	1	1
** Visual deficit**	2	1	1	0
** Dysphagia/dysarthria**	2	2	0	0
** Seizure**	1	1	0	0

SRS = Stereotactic radiosurgery.

Toxicity was classified using RTOG CNS toxicity criteria. [23] Grade 1 toxicity was observed in 3 (8.3%) patients, grade 2 in 1 (2.7%) patient and grade 3 in 1 (2.7%) patient respectively. Radiation necrosis, which is classified as grade 4 toxicity in this criteria developed in 1 (2.7%) patient. That patient underwent surgical treatment and radiation necrosis was pathologically confirmed. There were 4 patients suspicious of radiation necrosis by MR imaging and all of them underwent PET imaging. Other 3 patients showed true progression based on PET and these cases were not pathologically confirmed.

**Fig 1:**
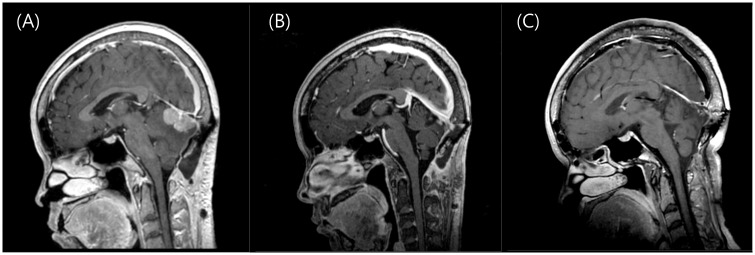
Illustrative cases of fractionated radiosurgery for large brain metastases. A 50-year-old woman was diagnosed with brain metastases 4 years after treatment for breast cancer. She underwent surgical removal at the time of diagnosis. (A) After 6 months she presented with progressive dysarthria, and follow-up MRI showed recurrence. Radiosurgery was performed because of the post-operative recurrence and tumor abutting the transverse sinus. The 16.5 cm^3^ cerebellar mass with was treated with a marginal dose of 8 Gy targeted to the 50% isodose line in 3 consecutive daily fractions. (B) One month after radiosurgery, the patient started systemic chemotherapy with Capecitabine, and the lesion dramatically decreased, and neurological symptoms also improved. (C) A final follow-up image obtained 27 months after radiosurgery shows that the lesion almost disappeared; the patient was still alive and on Gemcitabine and Cisplatin chemotherapy due to primary disease progression at the time of analysis, which was 30 months after SRS.

**Fig 2:**
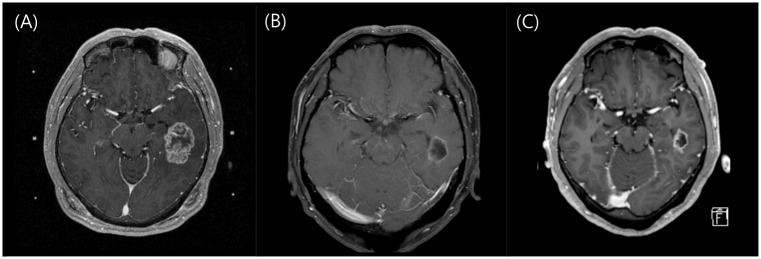
Another illustrative case of fractionated radiosurgery for large brain metastases. (A) A 53-year-old man was diagnosed with brain metastases on a staging work-up for non-small cell lung cancer. The lesion was 24.1 cm^3^; radiosurgery was performed with a marginal dose of 8 Gy targeted to the 50% isodose line in 3 consecutive daily fractions. The patient was also started on Gefitnib chemotherapy. (B) After 1 month, the lesion dramatically decreased to 4.9 cm^3^. (C) After 3 months, the lesion decreased to 3.4 cm^3^. The patient was later diagnosed with leptomeningeal carcinomatosis and was on Erlotinib chemotherapy at the time of analysis, which was 8 months after SRS.

Two representative cases are shown in Figs 1 and 2.

There was no significant difference in survival between single and multiple metastases (p = 0.73) and between those who had no previous treatment and those who had WBRT (p = 0.62). GPA scoring showed statistical significance related with survival (p = 0.002). Median survival according to the GPA score was 3.7 months for scores of 0–1, 17.9 months for scores of 1.5–2.5, and 23 months for scores of 3–4. Univariate analysis revealed that primary disease control (p = 0.001) and KPS score (p = 0.013) showed differences in survival outcomes. Lesions greater than 14 cm^3^ (3 cm in diameter), compared with smaller tumors, showed no significant differences in local control and overall survival. Multivariate analysis showed that control of primary disease (p = 0.001; HR 0.16, 95% CI 0.04–0.33) and KPS ≥70 (p = 0.011; HR 0.96, 95% CI 0.06–0.5) were significantly related to overall survival. The results of the statistical analyses are shown in Table 4.

**Table 4:** Prognostic factors related to overall survival of patients.

	Univariate		Multivariate		
Variable	HR	*P*	HR	*p*	95% CI
**Gender**	1.401	.118			
**Age ≥ 65 y**	1.030	.179			
**KPS ≥ 70**	0.218	.013	0.957	.011	0.055–0.501
**Control of primary disease**	0.344	.001	0.166	.001	0.043–0.326
**Extracranial metastases**	1.305	.294			
**Multiple metastases**	1.154	.732			
**Tumor volume > 14cm**^**3**^	0.996	.984			

HR = hazard ratio; CI = confidence interval; KPS = Karnofsky performance status.

## Discussion

This study was designed to assess the efficacy, side effects and overall survival of patients with large metastatic brain lesions treated by gamma knife multi-fraction SRS for three to four consecutive days using the same Leksell head frame. As described above, the treatment options for brain metastases include surgical resection, WBRT and SRS. Metastatic lesion size, location, multiplicity, comorbidity, and performance status are considered important factors when deciding which treatment options should be used.[6, 24, 25] Large metastases with mass effect and accessible location are good candidates for surgical resection. Lesions located in eloquent areas and patients in poor general condition are good candidates for radiosurgery; however, large size is a relative contraindication because of concerns regarding treatment-related toxicity increased radiation exposure.[12] Because the selection criterion for SRS was traditionally limited to lesions smaller than 3 cm, the treatment of choice for large metastases has typically been surgical resection. Occasionally, palliative WBRT or SRS was considered in inoperable cases.

Radiosurgery was originally defined as a conformal, single fraction of high dose radiation using a stereotactic method aimed to destroy target tissue while preserving adjacent normal tissue.[26] Local control is highly dependent on the radiation dose and is known to be less effective in single fraction doses lower than 15 Gy.[27, 28] The concept of radiosurgery has been expanded up to 5 fractions. The linear quadratic (LQ) model is used to calculate the biologic effective doses (BEDs) and to compare different tissues with different doses and fractionation.[18] The α/β ratio for metastatic brain tumors is estimated from 10 to 20, and a high α/β ratio indicates increased sensitivity to multiple-session treatments.[29–31]

Studies of single fraction SRS as a primary or salvage treatment for large brain metastases have been reported in the literature. Clinical outcomes such as overall survival and local control were favorable, but the treatment-related toxicity was considerable. Lee et al. treated 109 patients with a median dose of 18.0 Gy for a median tumor volume of 16.8 cm^3^, with progressive peritumoral edema in 19 (16.0%) patients.[32] Han et al. administered a single dose of 10–16 Gy to lesions with a mean volume of 22.4 cm^3^, and their results found unacceptable CNS toxicity of 18.8%. These authors suggested that a marginal dose of 11–12 Gy might be tolerable due to the high rate of radiation toxicities.[33] CNS toxicity has diverse manifestations ranging from mild, acceptable neurological symptoms to radiation necrosis and neurological deaths. The most significant risk factor for radiation necrosis is a high radiation dose in a large volume because the high dose eventually gives a high dose to the normal tissue in the periphery. The RTOG protocol 90–05 reported that lesions with a maximal diameter of 3 cm or more should use a maximal tolerated dose of 15 Gy due to toxicity.[23] Minniti et al. showed that a dose of 12 Gy in lesions larger than 8.5 cm^3^ carries a risk of radiation necrosis of 10% or higher, and the actuarial risk at 1 year was 24% for lesions 6.0–10.9 cm^3^ and 51% for those >10.9 cm^3^.[34] In this study as in Table 2, BED ranges from 24.2–60 and SFED ranges from 14.6–26.4 Gy. Compared with previous single fraction studies, it can be suggested that hypofractionation radiosurgery has similar efficacy and paucity of treatment related toxicity.

The significance of this study is the use of a daily fractionation method that was not previously reported in the literature. In fact, Gamma Knife-based SRS fractionated with intervals has been reported for large metastatic lesions.[16, 17] Higuchi et al. used a regimen of 30 Gy given in 3 fractions with a 2-week interval and showed promising results.[35] Yomo et al. performed two studies of fractionated Gamma Knife-based radiosurgery with 20 to 30 Gy at the 50% isodose line in two fractions, 3 to 4 weeks apart. One study of 27 patients with metastatic brain lesions reported a one-year local control rate of 61% and an overall survival rate of 45% with one patient (3.5%) experiencing radiation injury.[16] Another study of 58 patients with metastatic brain tumors reported a one-year local control rate of 64% and an overall survival of 47% with three patients (5.1%) having radiation injury.[17] The strategy of those fractionated GKS with an inter-fraction time of 2 to 4 weeks has the purpose of reducing tumor size so that the second treatment can be performed on a smaller volume more safely. In the current study, the overall survival rates at one and two years were 66.7 and 33.1%, respectively, and the medial survival time was 16.2 months. The local control rate was 90%, and radiation necrosis developed in one (2.7%). Compared to previous studies, our study produced good clinical outcomes with no significant difference in radiation-related toxicity; however, the interval and overall treatment periods were shorter in this study.[15–17, 36, 37] A daily consecutive treatment schedule is appropriate in terms of its good efficacy and tolerable toxicity rate. The placement of a stereotactic frame for several days on the patient had been our concern for this treatment; however, headache, pain or discomfort was tolerable. Another concern was moving of target between each fraction, and treatment location was confirmed by performing fusion of scout images.

Surgical resection is often used as a primary or adjuvant treatment option. Rapid decompression of mass effect is the strong merit of surgery in large tumors. However, surgery is difficult in some cases due to tumor size, the eloquent location of the lesion and perioperative complications. In eloquent area lesions, postoperative neurological deficit is a major concern. Obermueller et al. analyzed 56 patients with motor cortex metastatic lesions; 12 patients (21.4%) showed aggravated paresis, which remained permanently in 7 patients (12.5%).[38] Paek et al. reported surgical outcomes in a series of 208 patients with single or multiple metastases; the operative mortality rate was 1.9%, and neurological deterioration occurred in 13 patients (6%). There were also postoperative wound-related or systemic complications such as sepsis, pneumonia, or deep vein thrombosis in 21 patients (10%).[39] In the present study, there were no new neurologic deficits after the procedure, and pre-existing symptoms were mostly improved or at least stable, as shown in Table 3. Thus, it is difficult to conclude that radiosurgery is superior to surgery in terms of efficacy, but it is definitely a safer method in terms of immediate perioperative complications.

SRS to the resection cavity as an adjuvant to surgery is another treatment option. The purpose of this method is to improve the local control rate and decrease the need for WBRT. Several reports showed satisfactory outcomes, including in terms of local control. [40–43] Compared with the surgery-only group, the adjuvant SRS group had a significantly lower local failure rate; it can therefore be assumed that SRS is beneficial for local control.[42] One study used 9 Gy in 3 fractions to resection cavities larger than 3 cm in diameter and also showed good local control and low toxicity rates.[40] In our study, fractionated SRS was used as the primary treatment option, and the local control rate was similar to that of surgery plus adjuvant SRS. In patients with an inoperable condition or lesion, fractionated SRS as a primary treatment option should be considered.

LINAC-based, Cyberknife-fractionated radiosurgery has also been reported in many studies. The results show a local control of 70 to 96%, a median survival of 7 to 13.5 months and a radiation-related side effect rate of 5–10%.[8–11] Inoue et al. performed fractionation with Cyberknife, and the marginal dose ranged from 25–40 Gy with 50–75% isodose line.[44] The advantage of the Gamma Knife method allows a steeper dose fall-off so that a lower marginal dose can be prescribed with easier treatment planning compared to LINAC-based methods, which can help spare normal structures.[45]

This study was conducted in a retrospective manner with small number of cases with heterogeneous patients. Correlation of toxicity between single and hypofractionation may be not clear because this study does not have control group and have small number of cases. RTOG toxicity criteria was used for evaluation of treatment related toxicity but use of other QOL instruments and neurocognitive assessment should have been used for more accurate interpretation of toxicity which is also another limitation. We did not perform statistical comparison of various regimens. The optimal dose regimen is still unknown. Future prospective studies with large patient populations are needed to conclude that it is a safe and effective method of treatment. This study should serve as basis for prospective study.

## Conclusion

In this study, we demonstrated the efficacy and safety of hypofractionated Gamma Knife radiosurgery to treat large metastatic brain tumors or lesions located in the eloquent areas for three consecutive days with the same Leksell head frame. This result suggests that consecutive hypofractionated Gamma Knife SRS could be applied to large metastatic brain tumors with effective tumor control and low toxicity rates.
